# Immunotherapies Targeting Fish Mucosal Immunity – Current Knowledge and Future Perspectives

**DOI:** 10.3389/fimmu.2015.00643

**Published:** 2016-01-05

**Authors:** Shunsuke Koshio

**Affiliations:** ^1^Faculty of Fisheries, Kagoshima University, Kagoshima, Japan

**Keywords:** skin mucus, immunostimulant, functional feed, aquaculture, immunity

## Abstract

In recent years, studies on the mucosal immunity in fish species have shown much progress. Although there are some organs such as skin, gills, and gut are directly associated with the mucosal immunity of fish species, this mini review emphasizes the general knowledge on the role and production figures of skin mucus and factors affecting the secretion of skin mucus of fish species. As the skin mucus of fish species is the first defense line for protection against invading microorganisms such as pathogens (bacteria, virus), parasites, etc., the information for understanding the roles of the skin mucus is very important. Furthermore, the information in the review will shed light on the development of high quality aquafeeds for the sustainable aquaculture field as well.

## General Introduction

In fish mucosal immunity, the innate components of the immune system are the first barrier by which the host fish are protected from the attack of microorganisms. Gut, skin, and gills in fish species are the major mucosal surfaces and immune barriers. The mucus is one of the most important components for fish mucosal immunity. In adaptive immunity, on the other hand, there are many important components such as immunoglobulins (Igs), B and T lymphocytes, etc. [see the review by Gomez et al. (1)]. In this review, recent knowledge and findings on fish skin mucus are focused.

The presence and importance of innate immune parameters in the epidermal mucus on fish is well documented in the past (2, 3). It has been found that the epidermal layer of the skin is important because it secretes the mucus in addition to providing physical protection. The skin mucus plays a key role as the first defense line for protection in aquatic animals (2–5), and fish epidermal mucus contains numerous innate immune components such as glycoproteins, lysozyme, complement proteins, lectins, C-reactive proteins, flavoenzymes, proteolytic enzymes, and antimicrobial peptides as well as immunoglobulins (3, 6–9).

The external constituent of this barrier is a mucous gel that forms a layer of adherent mucus covering the epithelial cells (10) and is secreted by various epidermal or epithelial mucus cells (7). The fish skin mucus is mainly composed of water and glycoproteins (11), containing a large amount of high molecular weight oligosaccharides, namely mucins (12, 13). Mucin is one of the most important components in fish mucus. Previously, it was found that there are two structurally separate groups of mucins such as large secreted gel-forming mucins and membrane-bound forms (14). Recently, Perez-Sanchez et al. (15) challenged in the study of gilthead sea bream, *Sparus aurata*, to clarify mucin gene family, the tissue-specific expression pattern of mucins, and to determine whether mucins were altered by nutritional conditions and parasite infections. They identified six sequences, such as intestinal mucin (I-Muc), mucin2 (Muc2), mucin2-like (Muc2-like), mucin13 (Muc13), mucin18 (Muc18), and mucin 19 (Muc19), in which I-Muc, Muc13, and Muc18 are the members of membrane-bound-mucins, and Muc2, Muc2-like, and Muc19 are of secreted gel-forming mucins. Furthermore, the mucin gene expression pattern was tissue specific with a relatively low expression level in skin, gills, and stomach, respectively, but Muc18 is a major mucin in fish skin (15). According to the study of Van der Marel et al. (16) on carp, the mucin5B was mostly expressed in the skin, and its expression was up-regulated when β-glucan was administered.

It has been demonstrated that fish skin mucus is involved in the respiration, osmoregulation, reproduction, locomotion, defense against microbial infections, disease resistance and protection, etc. (7, 17). Fish skin mucus is continuously produced, and the structure and/or functions prevent the pathogen adherence to the underlying tissues and provide a medium in which antibacterial mechanisms may act (8). In a study on salmonids, Roberts and Powell (18) found that Atlantic salmon and brown trout responded to amoebic gill disease with a whole-body mucus, but rainbow trout did only with a gill mucus.

Recently, comparative studies on fish mucus for both freshwater and marine species were reported (8, 9). Five freshwater species such as mrigal, rohu, catla, rita, and spotted snakehead were used, and the components in mucus, namely lysozyme, proteases, phosphatases, esterase, and sialic acid were investigated in the study (8). The study indicated that enzyme activity depended on the species and/or habitat environment. For example, the values were high in mrigal and snakehead, but low in rohu and catla, which are relatively distributed in clean water. On the other hand, the levels of all factors measured in rita was found to be low. Serine and metalloproteases were the major mucus proteases in all fish species in the study. In marine species, five species such as gilthead seabream, European sea bass, shi drum, common dentex, and dusky grouper were investigated (9). The study showed that skin mucus contains *N*-acetylneuraminic acid, glucose, *N*-acetyl-glucosamine, *N*-acetyl-galactosamine, galactose, and fucose residues. It was also indicated that although IgM and lysozyme activity were very similar among species tested, protease, antiprotease, alkaline phosphatase, esterase, and peroxidase activities varied depending on the fish species tested. The mucus of gilthead seabream revealed high bactericidal effects against pathogenic bacteria not for the non-pathogenic ones.

To characterize the mechanisms of mucus as the first-line defense against pathogens, the study of Guardiola et al. (19) demonstrated that gilthead seabream skin mucus contained lower contents of IgM, similar level of lysozyme alkaline phosphatase and proteases, and higher level of esterase, peroxidase and antiprotease activities than those in serum. And the skin mucus revealed stronger bactericidal activity than the serum activity. Furthermore, a recent study on Atlantic salmon against salmon louse indicated difference in skin immune responses between the selected families (20) although increased mucus secretion by the Atlantic salmon when infected by salmon lice has been indicated (21). The study of Holm et al. (20) suggested that the ability to resistance against salmon lice depends on avoiding immunosuppression and not as much on the physical tissue barrier functions, but they assumed that increased mucous secretion by the Atlantic salmon might be important for parasite survival as nutritive elements for developing lice.

## Factors Affect the Mucus Production in Fish

To produce healthy cultured aquatic species is one of the most important and not so easy tasks for fish farmers with the sustainable operation. To achieve this, it is needed to promote the immunological responses of aquatic species under culture conditions. Therefore, it would be very important to know the role and efficient action of fish skin mucus. In recent years, there were several studies on the effects of functional substances and micronutrients with measuring mucus production and status.

Those like probiotic bacteria, oligosaccharide, β-glucan, etc. have been tested on mucus production (22–29). Epidermal mucus was enhanced by intake of lactic acid bacteria in Atlantic salmon (22) and mannan oligosaccharide in sea bass (24). Rodriguez-Estrada et al. (25) showed that the mucus production increased in rainbow trout-fed diet-containing inactivated *Enterococcus faecalis* or mannan oligosaccharide or a combination of both. Hoseinifar et al. (26) indicated in a study on freshwater swordtail that *Lactobacillus acidophilus* as feed supplement was effective on enhancing antibacterial activity of skin mucus, and the skin mucus protein level and alkaline phosphatase activity were also higher in *Lactobacillus* fed groups. They suggested that the recommended inclusion level was 6 × 10^8^ CFU/g. Recently, feeding trials were conducted to determine the effects of heat-killed *Lactobacillus plantarum* (HK-LP), β-glucan, and inactivated *Pediococcus pentosaceus* on immunological responses as well as growth performances of marine fish (27–29). Mucus secretion of red sea bream fed with all diet-containing HK-LP was higher than that fed with a HK-LP-free diet, and the value from a diet with 1000 ppm HK-LP concentration was significantly higher than that from a HK-LP-free diet (27). Dawood et al. (28) demonstrated that mucus secretion of red sea bream was significantly affected by either HK-LP or β-glucan, but they did not affect mucus bactericidal activity (Table 1). Relative amount of mucus of red sea ream fed with a diet containing 1000 ppm HK-LP together with 1000 ppm β-glucan was significantly higher than that with the basal diet. It was also found in their study that the mucus lysozyme activity significantly increased with increased HK-LP levels without β-glucan supplement while with β-glucan it did not change.

**Table 1:** **Mucus status of red sea bream fed diets containing different levels of heat-killed *Lactobacillus plantarum* (HK-LP) and β-glucan (BG)**.*

HK-LP (ppm)	BG (ppm)	LA(unit/ml)	BA (10^5^ CFU/ml)	Total amounts (relative value)
0	0	31.6^a^	5.71	1.00^a^
250	0	37.0^ab^	5.69	1.07^ab^
500	0	37.9^b^	5.34	1.02^ab^
1000	0	38.7^b^	6.67	1.12^ab^
250	1000	42.3^b^	6.19	1.08^ab^
500	1000	42.8^b^	7.47	1.10^ab^
1000	1000	40.5^b^	6.70	1.15^b^

*Within a column, values with different letters are significantly different (*P* < 0.05).

*LA, lysozyme activity; BA, bactericidal activity*.

In a *Pediococcus* study, Dawood et al. (29) found that the lysozyme activity in mucus of red sea bream was affected by the concentrations of inactivated *Pediococcus pentosaceus*, and the lysozyme activity was significantly higher in fish fed with a diet containing 1.6 × 10^12^ concentration than in fish fed with the basal diet (Table 2). Furthermore, mucus was significantly more secreted in *Pediococcus pentosaceus*-fed groups.

**Table 2:** **Mucus status of red sea bream fed with diets containing different concentrations of inactivated *Pediococcus pentosaceus***.*

Concentration (cells/g)	LA (unit/ml)	Total amounts (relative value)
0	32.5^a^	1.00^a^
1.6 × 10^10^	37.1^ab^	1.41^b^
1.6 × 10^11^	34.6^a^	1.42^b^
1.6 × 10^12^	51.3^b^	1.40^b^
3.2 × 10^12^	40.8^ab^	1.51^b^

*Within a column, values with different letters are significantly different (*P* < 0.05).

*LA, lysozyme activity*.

Other than probiotic bacteria, some micronutrients like vitamins have been found to be effective for mucus secretion. When red sea bream was fed with a diet containing 325 ppm vitamin C, lysozyme activity of skin mucus seemed to increase compared to that fed a vitamin C-free diet (30). Ren et al. (31) indicated in a study on Japanese eel that fish fed with diets containing 762 ppm vitamin C showed significantly higher lysozyme activity and bactericidal activity of mucus than fish fed with a diet with 32 ppm vitamin C. Furthermore, the mucus bactericidal activity was further enhanced with supplementation of dietary lactoferrin. Furthermore, it was found in a study on Caspian roach that dietary vitamin C significantly elevated skin mucus alkaline phosphatase, protein levels, and antimicrobial activity compared to a vitamin C-free group (32). As a functional supplement, lactoferrin has been tested to improve the health status of aquatic animals. Yokoyama et al. (33) demonstrated that mucus secretion significantly increased in spotted grouper when fed with diets containing lactoferrin from 400 to 1200 ppm compared to that of fish fed with a lactoferrin-free diet and concluded that oral lactoferrin administration could be an effective method to improve natural barriers of finfish.

In conclusion, since the skin mucus plays a key role as the first-defense line for protection of aquatic animals, controlled skin mucus secretion is very important for them to improve the survival, particularly for aquacultured species, leading to the fact that important components in mucus also increase. Although several dietary materials induce the increase of mucus secretion in aquatic animals, the effects against parasites are still under investigation. On the other hand, when aquatic animals are under stress conditions, the mucus secretion will also increase. Thus, the difference of mucus production between normal and stressed conditions should be carefully investigated.

## Conflict of Interest Statement

The author declares that the research was conducted in the absence of any commercial or financial relationships that could be construed as a potential conflict of interest.

## References

[1] Gomez D Sunyer JO Salinas I The mucosal immune system of fish: the evolution of tolerating commensals while fighting pathogens. Fish Shellfish Immunol (2013) 35:1729–39. 24099804 10.1016/j.fsi.2013.09.032 PMC3963484

[2] Fast MD Sims DE Burka JF Mustafa A Ross NW Skin morphology and humoral non-specific defense parameters of mucus and plasma in rainbow trout, coho and Atlantic salmon. Comp Biochem Physiol A (2002) 132:645–57. 10.1016/S1095-6433(02)00109-5 12044774

[3] Subramanian S MacKinnon SL Ross NW. A comparative study on innate immune parameters in the epidermal mucus of various fish species. Comp Biochem Physiol B Biochem Mol Biol (2007) 148:256–63. 10.1016/j.cbpb.2007.06.003 17618153

[4] Subramanian S Ross NW MacKinnon SL Comparison of antimicrobial activity in the epidermal mucus extracts of fish. Comp Biochem Physiol B Biochem Mol Biol (2008) 150:85–92. 10.1016/j.cbpb.2008.01.011 18342561

[5] Subramanian S Ross NW MacKinnon SL Comparison of the biochemical composition of normal epidermal mucus and extruded slime of hagfish (*Myxine glutinousa* L.). Fish Shellfish Immunol (2008) 25:625–32. 10.1016/j.fsi.2008.08.012 18817881

[6] Alexander JB Ingram GA Noncellular nonspecific defense mechanisms of fish. Annu Rev Fish Dis (1992) 2:249–79. 10.1016/0959-8030(92)90066-7

[7] Shephard KL Functions for fish mucus. Rev Fish Biol Fish (1994) 4:401–29. 10.1007/BF00042888

[8] Nigam AK Kumari U Mittal S Mittal AK. Comparative analysis of innate immune parameters of the skin mucous secretions from certain freshwater teleosts, inhabiting different ecological niches. Fish Physiol Biochem (2012) 38:1245–56. 10.1007/s10695-012-9613-5 22350522

[9] Guardiola FA Cuesta A Abellan E Meseguer J Comparative analysis of the humoral immunity of skin mucus from several marine teleost fish. Fish Shellfish Immunol (2014) 40:24–31. 10.1016/j.fsi.2014.06.018 24972341

[10] Van der Marel M Caspari N Neuhaus H Meyer W Enss M-L Steinhagen D. Changes in skin mucus of common carp, *Cyprinus carpio* L., after exposure to water with a high bacterial load. J Fish Dis (2010) 33:431–9. 10.1111/j.1365-2761.2010.01140.x 20298445

[11] Fletcher TC Non-specific defense mechanisms of fish. Dev Comp Immunol (1982) 2:123–32.

[12] Strous GJ Dekker J. Mucin-type glycoproteins. Crit Rev Biochem Mol Biol (1992) 27:57–92. 10.3109/10409239209082559 1727693

[13] Perez-Vilar J Hill R The structure and assembly of secreted mucins. J Biol Chem (1999) 274:31751–4. 10.1074/jbc.274.45.31751 10542193

[14] Moniaux N Escande F Porchet N Aubert JP Batra SK Structural organization and classification of the human mucin genes. Front Biosci (2001) 6:D1192–206. 11578969 10.2741/moniaux

[15] Perez-Sanchez J Estensoro I Redondo MJ Calduch-Giner JA Kaushik S Sitja-Bobadilla A. Mucins as diagnostic and prognostic biomarkers in a fish-parasite model: transcriptional and functional analysis. PLoS One (2013) 8:e65457. 10.1371/journal.pone.0065457 23776483 PMC3680472

[16] Van der Marel M Adamek M Gonzalez SF Frost P Rombout JHWM Wiegertjes GF Molecular cloning and expression of two β-defensin and two mucin genes n common carp (*Cyprinus carpio* L) and their up-regulation after β-glucan feeding. Fish Shellfish Immunol (2012) 32:494–501. 10.1016/j.fsi.2011.12.008 22227003

[17] Khong HK Kuah MK Jaya-Ram A Shu-Chien AC Prolactin receptor mRNA is up-regulated in discus fish (*Shmphysodon aequifasciata*) skin during parental phase. Comp Biochem Physiol B Biochem Mol Biol (2009) 153:18–28. 10.1016/j.cbpb.2009.01.005 19272315

[18] Roberts SD Powell MD. The viscosity and glycoprotein biochemistry of salmonid mucus varies with species, salinity and the presence of amoebic gill disease. J Comp Physiol B (2005) 175:1–11. 10.1007/s00360-005-0473-5 15517284

[19] Guardiola FA Cuesta A Arizcum M Meseguer J Esteban MA Comparative skin mucus and serum humoral defense mechanisms in the teleost gilthead seabream (*Sparus aurata*). Fish Shellfish Immunol (2014) 36:545–51. 10.1016/j.fsi.2014.01.001 24412437

[20] Holm H Santi N Kjøglum S Perisic N Skugor S Evensen Ø. Difference in skin immune responses to infection with salmon louse (*Lepeophtheirus salmonis*) in Atlantic salmon (*Salmo salar* L.) of families selected for resistance and susceptibility. Fish Shellfish Immunol (2015) 42:384–94. 10.1016/j.fsi.2014.10.038 25449368

[21] Fast MD Ross NW Mustafa A Sims DE Johnson SC Conboy GA Susceptibility of rainbow trout *Oncorhynchus mykiss*, AAtlantic salmon *Salmo salar* and Coho salmon *Oncorhynchus kisutch* to experimental infection with sea lice *Lepeophtheirus salmonis*. Dis Aquat Organ (2002) 52:57–68. 10.3354/dao052057 12517006

[22] Salinas L Myklebust R Esteban MA Olsen RE Meseguer J Ringo E. *In vitro* studies of *Lactobacillus delbrueckii* subsp. *lactis* in Atlantic salmon (*Salmo salar* L.) foregut: tissue responses and evidence of protection against *Aeromonas salmonicida* subsp. *salmonicida* epithelial damage. Vet Microbiol (2008) 128:167–77. 10.1016/j.vetmic.2007.10.011 18054448

[23] Sweetman JW Torrecillas S Dimitroglou A Rider S Davies SJ Izquierdo MS Enhancing the natural defenses and barrier protection of aquaculture species. Aquac Res (2010) 41:345–55. 10.1111/j.1365-2109.2009.02196.x

[24] Torrecillas S Makol A Caballero MJ Montero D Gines R Sweetman J Improved feed utilization, intestinal mucus production and immune ­parameters in sea bass (*Dicentrarchus labrax*) fed mannan oligosaccharides (MOS). Aquac Nutr (2011) 17:223–33. 10.1111/j.1365-2095.2009.00730.x

[25] Rodriguez-Estrada U Satoh S Haga Y Fushimi H Sweetman J Effects of inactivated *Enterococcus faecalis* and mannan oligosaccharide and their combination on growth, immunity, and disease protection in rainbow trout. North Am J Aquacul (2013) 75:416–28.

[26] Hoseinifar SH Roosta Z Hajimoradloo A Vakili F The effects of *Lactobacillus acidophilus* as feed supplement on skin mucosal immune parameters, intestinal microbiota, stress resistance and growth performance of black swordtail (*Xiphophrus helleri*). Fish Shellfish Immunol (2015) 42:533–8. 10.1016/j.fsi.2014.12.003 25514375

[27] Dawood MAO Koshio S Ishikawa M Yokoyama S Effects of heat killed *Lactobacillus plantarum* (LP20) supplemental diets on growth performance, stress resistance and immune response of red sea bream, *Pagrus major*. Aquac Nutr (2015) 442:29–36. 10.1016/j.aquaculture.2015.02.005

[28] Dawood MAO Koshio S Ishikawa M Yokoyama S Interactive effects of dietary supplementation of heat-killed Lactobacillus plantarum and β-glucan on growth performance, digestibility and immune response of juvenile red sea bream, Pagrus major. Fish Shellfish Immunol (2015) 45:33–42. 10.1016/j.fsi.2015.01.033 25661844

[29] Dawood MAO Koshio S Ishikawa M Yokoyama S Effects of dietary inactivated *Pediococcus pentosaceus* on growth performance, feed utilization and blood characteristics of red sea bream, *Pagrus major* juvenile. Aquacul Nutr (2015). 10.1111/anu.12314

[30] Ren T Koshio S Uyan O Komilus CF Yokoyama S Ishikawa M Effects of dietary vitamin C on blood chemistry and nonspecific immune response of juvenile red sea bream, *Pagrus major*. J World Aquac Soc (2008) 39:797–803. 10.1111/j.1749-7345.2008.00216.x

[31] Ren T Koshio S Ishikawa M Yokoyama S Micheal FR Uyan O Influence of dietary vitamin C and boivine lactoferrin on blood chemistry and non-specific immune responses of Japanese eel, *Anguilla japonica*. Aquac Nutr (2007) 267:31–7. 10.1016/j.aquaculture.2007.03.033

[32] Roosta Z Hajimoradloo A Ghorbani R Hoseinifar SH. The effects of dietary vitamin C on mucosal immune responses and growth performance in Caspian roach (*Rutilus rutilus* caspicus) fry. Fish Physiol Biochem (2014) 40:1601–7. 10.1007/s10695-014-9951-6 24965492

[33] Yokoyama S Koshio S Takakura N Oshida K Ishikawa M Gallardo-Cigarroa FJ Effect of dietary bovine lactoferrin on growth response, tolerance to air exposure and low salinity stress conditions in orange spotted grouper *Epinephelus coioides*. Aquac Nutr (2006) 255:507–13. 10.1016/j.aquaculture.2005.12.001

